# Pre-auricular Subtemporal Approach for Intracranial Angiofibroma

**DOI:** 10.22038/IJORL.2024.74214.3496

**Published:** 2024-03

**Authors:** Neizekhotuo Brian Shunyu, Zareen Lynrah, Manu C balakrishnan, Lham Dorjee, Ratan Medhi

**Affiliations:** 1 *Department of Otorhinolaryngology Head and Neck Surgery, AIIMS, Guwahati, India.*; 2 *Department of Otorhinolaryngology Head and Neck Surgery, NEIGRIHMS, Shillong, Meghalaya, India.*

**Keywords:** Angiofibroma, Intracranial, Pre-auricular, Lateral subtemporal

## Abstract

**Introduction:**

In around 10-20% of angiofibroma cases, the tumor penetrates the skull base to involve intracranial structures, posing difficulty in treating them surgically. Today, advancement in skull base surgery has brought about a paradigm shift, and extensive angiofibroma tumors with intracranial extension are approached surgically today with minimal morbidity.

**Materials and Methods:**

This study was a retrospective analysis of angiofibroma with significant intracranial extension Radkowski staging IIIb from 2011 to 2021 who came to our center. There were seven children of angiofibroma with significant intracranial extension Radkowski staging IIIb, out of whom, four patients had undergone surgical resection at our center. Three patients underwent surgery by pre-auricular lateral subtemporal approach and one patient by maxillary swing approach. Preoperative embolization was done in all the patients one day before the day of operation.

**Results:**

Gross total removal of the tumor was achieved in all three patients who had undergone pre-auricular lateral subtemporal approach with no permanent complication. All three patients had a minimum follow-up of one year with no recurrence.

**Conclusion:**

The pre-auricular lateral subtemporal approach provides the shortest shallow route to the affected skull base with direct visualization of the tumor base. Hence recommended for angiofibroma with Radkowski staging IIIb.

## Introduction

Angiofibromas, also known as Juvenile Nasopharyngeal Angiofibromas (JNA) are benign but locally aggressive highly vascular tumors. Various studies suggested that the origin of JNA is from the superior margin of the sphenopalatine foramen, while some thought it originated from the pterygoid canal (1,2). Though histologically benign, JNA causes significant morbidity and occasionally mortality as they aggressively spread submucosally to adjacent structures (3). After its origin from the sphenopalatine foramen, it extends either laterally through the pterygopalatine fossa into the infratemporal fossa or medially into the nasopharynx. The usual way of tumor spreads intracranially is from the pterygopalatine fossa via the superior orbital fissure or from the infratemporal fossa via the inferior orbital fissure into orbit and via the superior orbital fissure into the para-sellar region or cavernous sinus of the middle cranial fossa. Medially, the tumor grows into the nasopharynx. From the nasopharynx, the tumor can expand superiorly eroding the sphenoid sinus and can involve the pituitary fossa medially to the cavernous sinus by direct erosion of the superior wall of the sphenoid sinus. Enlarging tumors can also spread along the skull base posterior to the pterygoid process and the para-pharyngeal space. Also, from the pterygoid canal, the cancellous bone of the pterygoid base and the greater wing of the sphenoid can be invaded, resulting in an invasion of the middle cranial fossa lateral to the cavernous sinus. On rare occasions, from the infratemporal fossa, the tumor can invade the middle cranial fossa through direct erosion of the floor or the foramen rotundum and rarely from the foramen ovale. The tumor can also extend into the anterior cranial fossa by eroding the roof of the ethmoid sinus. 

In around 10-20% of JNA cases, the tumor penetrates the skull base to involve intracranial structures, posing difficulty in treating them surgically (4). Previously, these seemingly unresectable intracranial tumors were subjected to radiotherapy or incomplete resection of the tumor (5). Of course, radiotherapy has some success in terms of tumor regression and symptom relief (6,7). However, the use of radiotherapy, especially in the pediatric age group, has serious consequences like secondary malignancies, cranial-neuropathy, brainstem compression, pituitary dysfunction, cataracts, osteoradionecrosis, growth arrest, etc. (6,8). Today, advancement in skull base surgery has brought about a paradigm shift away from radiotherapy in the management of JNA with intracranial extension, which otherwise was thought earlier to be inoperable. Thus, extensive JNA tumors with intracranial extension are approached surgically today with minimal morbidity. Many surgical approaches described in the literature to remove these tumors indirectly point to the complexity of removing these tumors, as this is a region of difficult exposure with complex anatomical structures. The main aim of all these approaches is to get maximum exposure and vascular control with minimal associated morbidity.

## Results

Gross total removal of the tumor was achieved in all three patients who had undergone a pre-auricular lateral subtemporal approach. Pre-operative contrast-enhanced computed tomography (CECT) scan, showing tumor involvement of the cavernous sinus (Figure 5). 

**Fig 1:**
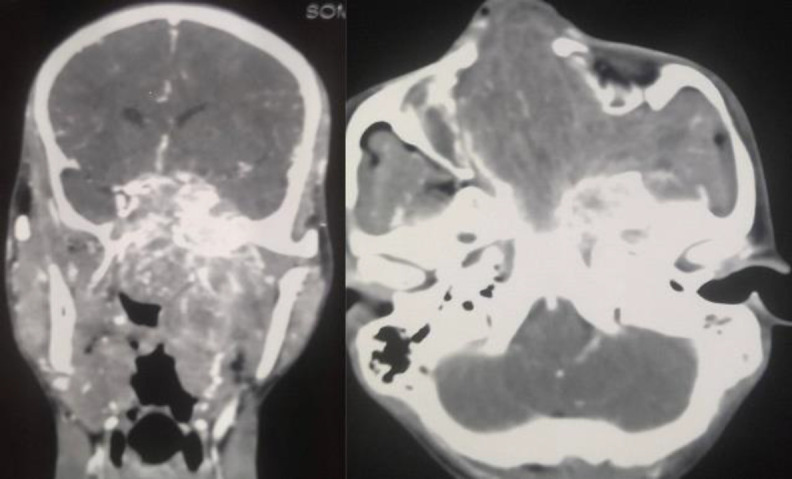
Pre-operative CECT scan

**Fig 2:**
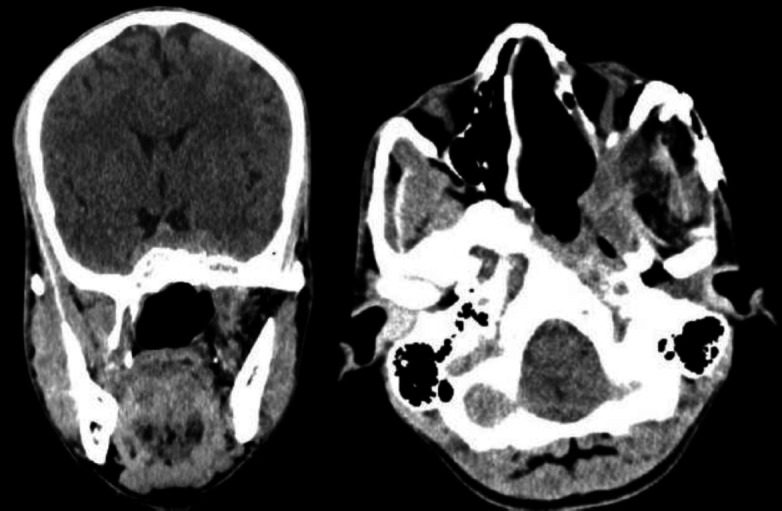
Post-operative CECT scan

**Fig 3:**
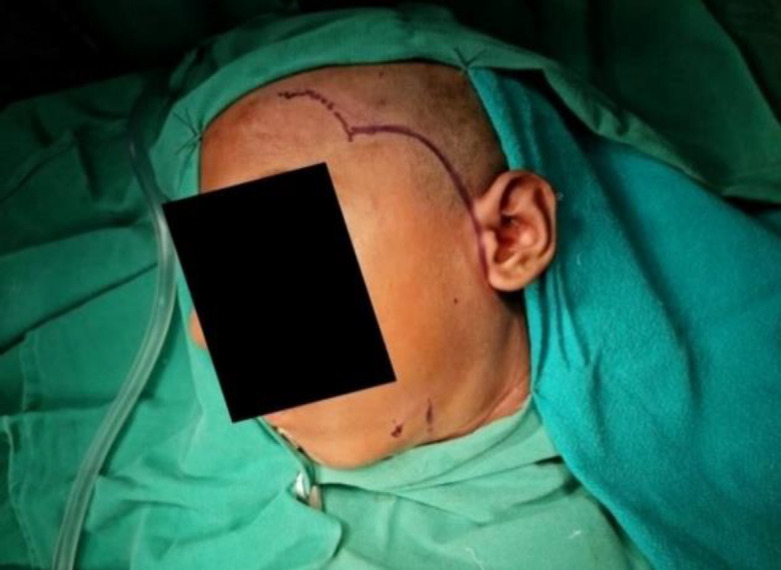
Modified skin Incision

**Fig 4:**
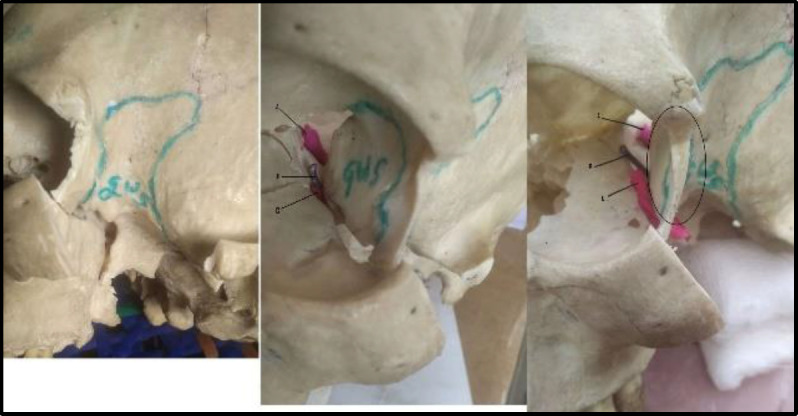
Dry skull base bone

**Fig 5:**
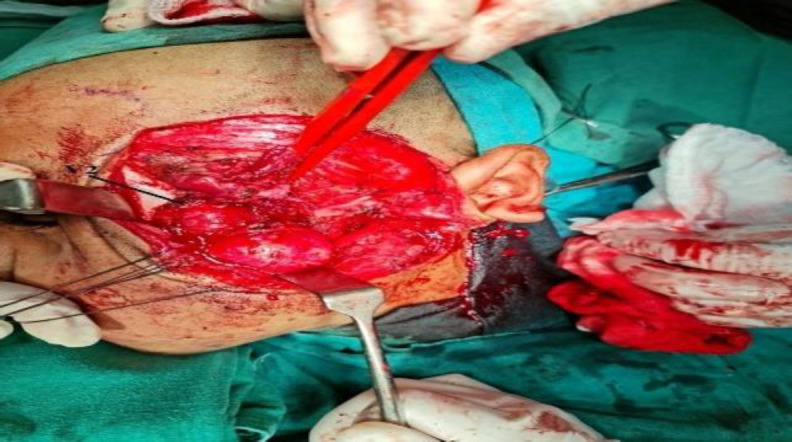
Tumor being pulled out through the laterally created defect and V2 seen over the tumor surface

**Fig 6:**
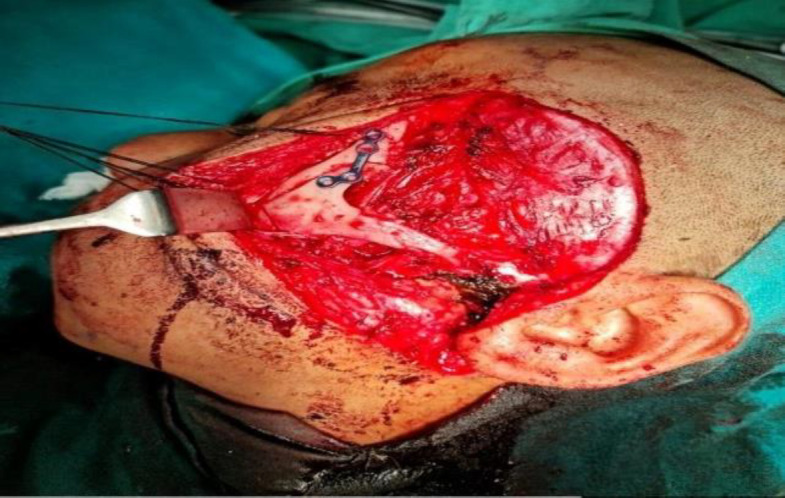
Zygomatic arch and bone being fixed back

This patient with cavernous involvement had significant blood loss. Intra-operatively during removal of the cavernous component of the tumor, there was profuse venous bleeding. The bleeding area was packed and compressed with cottonoid. Finally, the bleeding was controlled with surgicel and fibrin sealant (Evicel). In this patient, approximately 1500 ml of blood was lost. Four units of whole blood were transfused to the patient intraoperatively and later in the intensive care unit (ICU) two more units of blood were transfused to the patient. The patient did not have any permanent complications. In the rest, two patients' blood loss was below 700 ml, and only one unit of blood was transfused to each patient. The duration of the operation for the three patients was 8 hours 20 minutes (first patient), 7 hours 10 minutes (second patient), and 7 hours 50 minutes (third patient with cavernous involvement) with an average ICU stay of 2.3 days. There was no cerebrospinal fluid (CSF) leakage in any patient. There was a temporary trismus in two patients. On examination, cheek numbness was present in all the patients, which otherwise was not complained by the patients. No patients had forehead weakness. The first and second patients had followed up for 36 and 21 months respectively while the patient with cavernous involvement had completed 14 months of follow-up. All three patients had no recurrence of the tumor. The post-operative CECT scan of the third patient showed no residual tumor (Figure 6).

## Discussion

Historically, angiofibroma with intracranial extension has been associated with an increased rate of uncontrolled hemorrhage, neurological deficits, and recurrence (9). Previously, tumors especially those invading the cavernous sinus were thought to be inoperable given injury to the internal carotid artery, bleeding from the cavernous venous plexus, cranial nerves injury, or uncontrollable CSF leak (10). Today, though, advancement in skull base surgery has brought about a paradigm shift; surgical management of JNA with intracranial extension remains a major surgical challenge to a skull base surgeon. Angiofibroma is a rare tumor and accounts for 0.05 percent of all head and neck neoplasms. Its incidence has been stated to be as low as 1/50,000 new otolaryngological patients (11). Thus, the frequency of angiofibroma with significant intracranial extension will be quite low even in high-volume centers. Hence selection of the surgical approach to these tumors should not only be based on the surgeon's experience along but also on the approaches that give an adequate visualization of the tumor and the easy ability to control the bleeding during the surgery should be considered. 

There had been a controversy existing in deciding the surgical approach for angiofibroma with intracranial extension. There were many surgical approaches described in the literature to remove these tumors. The maxillary swing approach has been described. The maxillary swing approach gives wide access to Nasopharynx, Pterygopalatine fossa, and pterygomaxillary fissure, but the approach is not adequate for angiofibroma with intracranial extension. The transzygomatic and trans palatal combined approach had also been described for large angiofibroma. This approach gives no facial scar but provides only limited exposure to the middle cranial fossa. The facial translocation procedure gives sufficient exposure to the middle cranial fossa, but the procedure requires facial incisions through modified Weber-Ferguson incisions. This procedure also requires the division of the temporal branch of the facial nerve and the sacrifice of the infraorbital nerve. The combined frontotemporal and lateral infratemporal fossa approach, as described by Mickey et al. (12), is used mostly by neurosurgeons to remove angiofibroma with significant intracranial extension. This approach gives excellent exposure and should be the preferred choice when there is angiofibroma with intradural intracranial extension. The mechanism of bone destruction of angiofibroma occurs because of the pressure effect of the tumor, rather than by infiltration. For this reason, intracranial extension is mostly extradural, with a very rare dural invasion (13,14). The pre-auricular lateral subtemporal approach described in this paper is similar to the combined frontotemporal and lateral infratemporal fossa approach in terms of approach but differs in terms of soft tissue dissection and bony removal. Unlike the combined frontotemporal and lateral infratemporal fossa approach, the pre-auricular lateral subtemporal approach is not associated with facial scars and craniofacial defects. As highlighted in Figure 2, the pre-auricular lateral subtemporal approach gives the shortest and the most direct access to the cranial base after the removal of the zygomatic arch together with part of the zygomatic bone forming the lateral orbital wall. This approach also gives direct visualization of the tumor base at the beginning of the dissection. The intracranial component of the tumor can be directly dissected. In this approach, the bone removal is performed as per requirement according to the extent of the tumor and is guided by the tumor. Depending upon the tumor extension, the bone can be removed further down the middle cranial fossa to expose the tumor involving the cavernous sinus. This is the advantage of this approach as we are guided by the tumor path of spread and this allows extracranial as well as intracranial parts of the tumor to be dissected in one plane. Since angiofibroma has a well-defined capsule with a cleavage plane, it can be removed by gentle traction and dissection after it gets well exposed. The pre-auricular incision runs through the temporal fossa and the exposure can be expanded as required, especially for extensive intracranial diseases. In this approach, we do subtemporal craniectomy and not craniotomy. 

Though the endoscopic endonasal approach is preferred for mild and moderately advanced JNA, but open approach or open approach endoscopic assisted still plays an important role in the treatment of advanced stage JNA, especially Radkowski staging IIIb. Angiofibroma tumors with cavernous sinus involvement are very challenging even for a senior skull base surgeon with potential complications like uncontrollable hemorrhage, cranial nerve injury, persistent cerebrospinal fluid leakage, and high incidence of residual tumor (15,16). One patient we operated on had cavernous sinus involvement, as shown in Figure 5. During the surgery, this patient had profuse venous bleeding from the cavernous sinus, which was finally controlled with Surgicel and Evicel. Evicel is a fibrin sealant hemostatic agent comprising two components, human clottable protein, and human thrombin. In our case, since there was significant oozing of venous blood even with surgicel packing, we injected Evicel over the surgicel. We found Evicel applied over the surgicel to be good at controlling venous bleeding from the cavernous sinus. As shown in Figure 2, after the removal of the required bone, this approach gives excellent exposure to the anterior part of the cavernous sinus. This approach gives the advantage of a shallow wide field after the removal of the zygomatic arch together with part of the zygomatic bone forming the lateral orbital wall as shown in figure 2. Getting a shallow wide field allows the surgeon to do a careful dissection of the intracranial portion of the tumor. Since this lateral approach provides a wide shallow field, a microscope can always be used if required. Guarded traction and diligent dissection are needed for the removal of the intracranial component, especially the cavernous component. There is no clear consensus in the literature regarding the surgical approach to angiofibroma with advanced intracranial or cavernous sinus involvement (17,18). Pre-auricular lateral subtemporal approach as described in this paper can be used for angiofibroma with limited cavernous sinus involvement. Pre-auricular lateral subtemporal approach gives direct exposure to the base of the pterygoid and drilling the base of the pterygoid perhaps probably explains the low rate of recurrence of the tumor with this approach. Our three patients with pre-auricular lateral subtemporal approach had no recurrence of the tumor, though all were Radkowski staging IIIb during the follow-up period ranging from 36 to 14 months; whereas the patient who underwent maxillary swing approach had recurrent of the tumor at the 7 months of follow-up. Aesthetically, there was no noticeable depression in the temporal fossa area as the temporalis muscle was rotated back into the infratemporal fossa cavity to provide soft tissue bulk in the cavity. Elevating the temporalis muscle from the coronoid process below is our modification, differing from the other earlier methods (19). 

With this modification, the temporalis muscle is easily rotated to protect the exposed dura, separating the exposed dura from the nasopharynx. The temporomandibular joint, parotid gland, and facial nerve, except the frontal branch, are left undisturbed with no visible scar in this approach. 

## Conclusion

The pre-auricular lateral subtemporal approach is a good approach for angiofibroma with intracranial extension as this approach provides the shortest shallow route to the affected skull base with direct visualization of the tumor base with the ease of controlling the bleeding during the surgery. Hence recommended for angiofibroma with Radkowski staging IIIb. 
